# Efficacy of integrating short-course chemotherapy with Chinese herbs to treat multi-drug resistant pulmonary tuberculosis in China: a study protocol

**DOI:** 10.1186/s40249-021-00913-5

**Published:** 2021-11-06

**Authors:** Shun-Xian Zhang, Lei Qiu, Cui Li, Wei Zhou, Li-Ming Tian, Hui-Yong Zhang, Zi-Feng Ma, Xian-Wei Wu, Xing Huang, Yu-Wei Jiang, Shao-Yan Zhang, Zhen-Hui Lu

**Affiliations:** 1 Longhua Hospital Shanghai University of Traditional Chinese Medicine, 725 South Wanping Road, Shanghai, 200032 People’s Republic of China; 2 Shanghai University of Traditional Chinese Medicine, 1200 Cai Lun road, Shanghai, 201203 People’s Republic of China; 3 Center for Energy Metabolism and Reproduction, Shenzhen Institute of Advanced Technology, Chinese Academy of Sciences, Shenzhen, 518055 China

**Keywords:** Pulmonary tuberculosis, Multidrug-resistant, Chemotherapeutic drug, Chinese herbs, Randomized controlled trial

## Abstract

**Background:**

Tuberculosis (TB) caused *Mycobacterium tuberculosis* (*M.tb*) is one of infectious disease that lead a large number of morbidity and mortality all over the world. Although no reliable evidence has been found, it is considered that combining chemotherapeutic drugs with Chinese herbs can significantly improves the cure rate and the clinical therapeutic effect.

**Methods:**

Multi-drug resistant pulmonary tuberculosis (MDR-PTB, *n* = 258) patients with Qi-yin deficiency syndrome will be randomly assigned into a treatment group (*n* = 172) or control/placebo group (*n* = 86). The treatment group will receive the chemotherapeutic drugs combined with Chinese herbs granules (1 + 3 granules), while the control group will receive the chemotherapeutic drugs combined with Chinese herbs placebo (1 + 3 placebo granules). In addition, MDR-PTB (*n* = 312) patients with Yin deficiency lung heat syndrome will be randomly assigned to a treatment (*n* = 208) or control/placebo (*n* = 104) group. The treatment group will receive the chemotherapeutic regimen combined with Chinese herbs granules (2 + 4 granules), while the control group will receive the chemotherapeutic drugs and Chinese herbs placebo (2 + 4 placebo granules). The primary outcome is cure rate, the secondary outcomes included time to sputum culture conversion, lesion absorption rate and cavity closure rate. BACTEC™ MGIT™ automated mycobacterial detection system will be used to evaluate the *M.tb* infection and drug resistance. Chi-square test and Cox regression will be conducted with SAS 9.4 Statistical software to analyze the data.

**Discussion:**

The treatment cycle for MDR-PTB using standardized modern medicine could cause lengthy substantial side effects. Chinese herbs have been used for many years to treat MDR-PTB, but are without high-quality evidence. Hence, it is unknown whether Chinese herbs enhances the clinical therapeutic effect of synthetic drugs for treating MDR-PTB. Therefore, this study will be conducted to evaluate the clinical therapeutic effect of combining Chinese herbs and chemotherapeutic drugs to treat MDR-PTB cases. It will assist in screening new therapeutic drugs and establishing treatment plan that aims to improve the clinical therapeutic effect for MDR-PTB patients.

***Trial registration*:**

This trial was registered at ClinicalTrials.gov (ChiCTR1900027720) on 24 November 2019 (prospective registered).

**Graphical Abstract:**

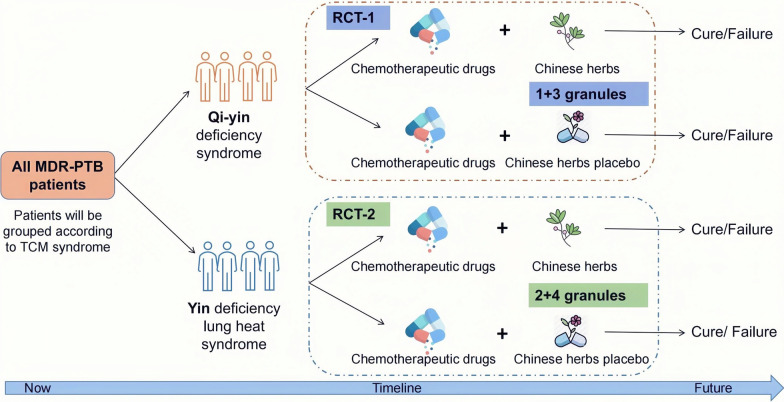

**Supplementary Information:**

The online version contains supplementary material available at 10.1186/s40249-021-00913-5.

## Background

Tuberculosis (TB) is a communicable disease caused by *Mycobacterium tuberculosis* (*M.tb*) and a major cause of ill health worldwide. It is one of the 10 most common causes of death and the leading cause of mortality by a single infectious agent [1]. More than 10 million individuals suffered from TB and 1.4 million deaths were occurred in 2019 globally [1]. The global incidence of TB is not sufficiently fast to decline to achieve the first milestone of the “End TB Strategy” due to drug-resistant TB (DR-TB) emergence [1]. Such resistant strains include rifampicin-resistant TB (RR-TB), multidrug-resistant TB (MDR-TB), and extensively drug-resistant TB (XDR-TB, Additional file 1: Appendix 1) [2]. DR-TB has created a global public health crisis, threatening the progress of “Ending global TB epidemics” by 2035 [3]. Globally in 2019, an estimated 3.3% of new cases and 18.0% of previously treated patients were MDR/RR-TB [1]. Overall, there were an estimated 465 000 MDR/RR-TB cases in 2019, of which 78.0% of RR-TB cases were MDR-TB. Of new MDR-TB patients, nearly 50.0% patients were in India (27.0%), China (14.0%) and the Russian Federation (8.0%) [1]. Approximately 15.0% MDR-TB cases died during treatment, 21.0% have unknown outcomes, while only 56.0% complete treatment worldwide [2].

China suffers the second-highest burden of MDR pulmonary TB (PTB) [1]. It has been estimated that 7.1% of new cases of PTB and 21.0% of previously-treated PTB cases in 2018 were MDR-PTB patients [1]. There were an estimated 58 000 MDR-PTB/RR-PTB cases annually, accounting for 10.0% of the global burden. However, only 22.5% of the MDR-PTB/RR-PTB cases were confirmed in the laboratory, while only 10.2% received treatment. This represents a considerably lower rate of detection and treatment coverage for MDR-PTB than the global average level [3, 4].

Over past decades, although the Chinese government has implemented control and treatment measures to control PTB, the situation has not substantially improved. Any achievements may have been compromised by the rising prevalence of DR-PTB. A treatment scheme for MDR-PTB patients will provide insights on policy and strategy development consistent with the “End TB Strategy” [1]. The treatment for MDR-PTB patients is either long therapeutic schedule (18 to 24 months) or short treatment plan (9 to 12 months) [5]. Long therapeutic schedule includes the third-generation fluoroquinolone and a second-line injectable combined with at least five anti-tuberculosis medications considered to be effective, based on drug susceptibility testing (DST), or at least four considered effective plus pyrazinamide (PZA or Z) [6]. Adverse events (AEs) are thought to be the most important clinical consideration in the longer regimen, such as elevated liver enzymes, gastrointestinal disorders, and ototoxicity [7]. More than half of MDR-PTB patients experience one or more AEs during treatment procession [8, 9]. The World Health Organization (WHO) treatment guidelines for RR/MDR-PTB recommend patients receive long oral regimens lasting 18–20 months, it strongly advising to combine bedaquiline (Bdq), levofloxacin (Lfx), or moxifloxacin (Mfx) with linezolid (Lzd) to treat MDR-PTB, supplemented with cycloserine (Cs) and/or clofazimine (Cfz). This long therapeutic schedule will result in predictable adverse reactions, because both Lzd and Cs are known for frequent serious AEs, while Bdq, Mfx and Cfz may excessively increase patient’s Q-T interval (the time from Q wave to the end of the T wave) [6]. It is challenging to monitor these expected AEs and their management in resource-limited settings may lead to frequent modifications and the provision of a less effective regimen.

Since 2016, the WHO guidelines have provided the option to treat RR/MDR-PTB with a standardized regimen with 9 to 12 months (“short regimen”) instead of an individualized regimen of at least 20 months (Additional file 1: Appendix 1) [10, 11]. Short regimens are defined as a standardized regimen that includes 4 to 6 months administration of kanamycin (Km), Mfx, prothionamide (Pto), Cfz, Z, ethambutol (EMB or E), and high-dose isoniazid (INH or I), followed by 5 to 8 months of Mfx, Cfz, PZA, EMB, optionally [12]. The following within-class drug substitutions are permitted, gatifloxacin (Gfx) or Lfx instead of Mfx, ethionamide (Eto) instead of Pto, and amikacin (Am) or capreomycin (Cm) instead of Km (Additional file 1: Appendix 1) [13]. The short regimen has been associated with a substantially smaller loss to follow-up during treatment compared with individualized long regimens [14], the results from WHO shown that the overall treatment success of short regimen was not inferior to long regimens [14].

Nowadays, a number of MDR-PTB patients may face that no alternative drug was used due to the DR-TB *M.tb* strains, the slow development of new drugs, and adverse reactions leaded chemical synthesized drugs. Confronted with this situation, Chinese herb may be an appropriate choice, Chinese herb has been used to treat PTB for more than 2000 years. Although the treatment of PTB with Chinese herbs began earlier than modern medicine [15], it has failed to control its prevalence over the past years. However, Chinese herb should not be overlooked in current serious situation of MDR-PTB prevalence, it may have some advantages for combatting MDR-PTB. Firstly, Chinese herbs are abundant and readily available. Secondly, herbs may reduce the side effects caused with chemical synthetic drugs, many patients suffer liver and kidney injury following the administration of chemotherapeutic drugs, which cannot be tolerated or ignored in the process of treating MDR-PTB. Thirdly, Chinese herbs can improve clinical symptoms caused by *M.tb*. Hence, the Chinese herbs in conjunction with chemical synthetic drugs to treat MDR-PTB may be one of optimal therapy.

Therefore, a hospital-based, large-scale, multi-center, parallel-group, double-blind, randomized controlled trial (RCT) was conceived and conducted to investigate the clinical therapeutic effect of MDR-PTB patients treated with chemotherapeutic drugs and Chinese herbs. New and more effective therapeutic schedule or drug for MDR-PTB may be obtained that improves the clinical therapy effects and health-related quality of life for the MDR-PTB patients.

## Objectives of the study

This RCT will be obtain several specific results. Firstly, the basic characteristics of MDR-PTB patients with Qi-yin deficiency syndrome or Yin deficiency lung heat will be obtained. Secondly, the risk factors for MDR-PTB will be explored by observational research. Thirdly, the factors affecting the MDR-PTB outcome will be identified in MDR-PTB cases. Fourthly, the efficacy of modern chemotherapeutic drugs combined with Chinese herbs will be obtained and appraised.

## Methods/design

### Study setting

**Fig. 1:**
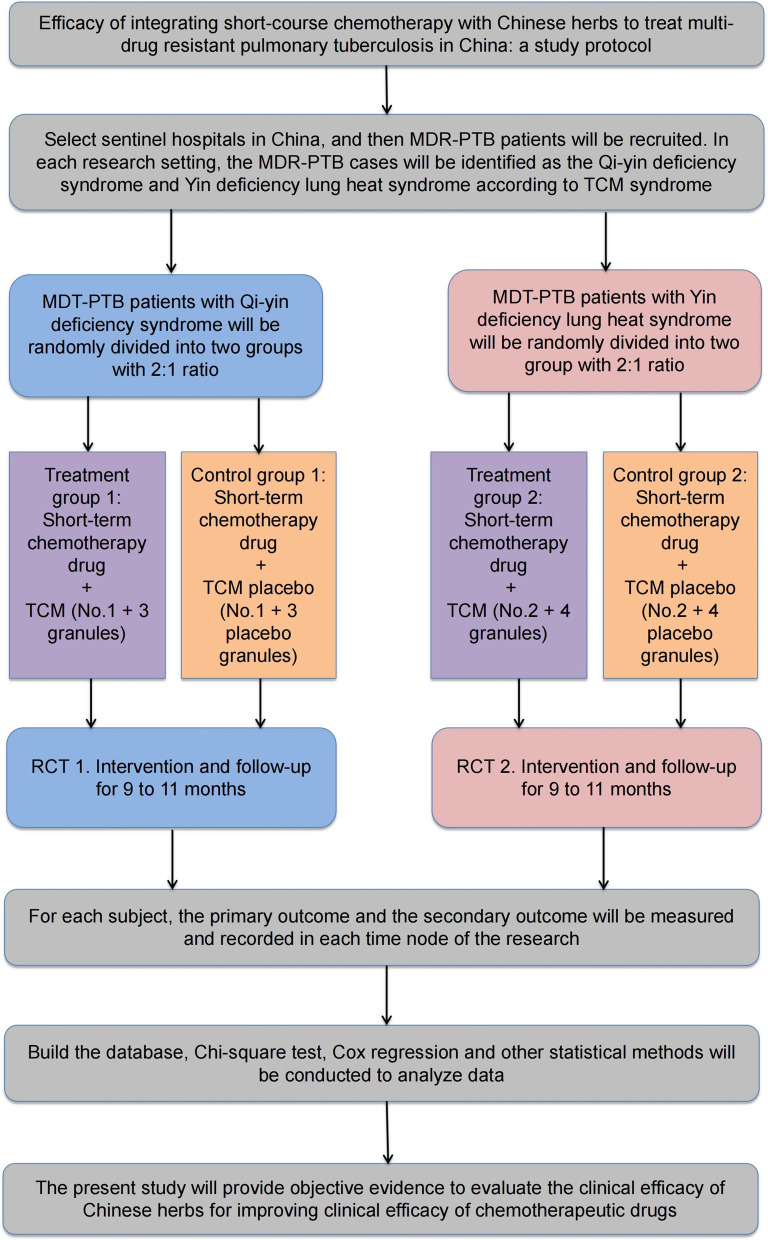
Flow diagram illustrating study design. *MDR-PTB* Mul-tidrug resistant pulmonary tuberculosis; *TCM* Traditional Chinese medicine.

In present study, 32 research centers (sentinel hospitals) were recruited from 23 provinces of China that have a high prevalence of MDR-PTB (Table 1, Fig. 1, Additional file 1: Appendix 1). These hospitals are qualified to treat MDR-PTB patients. Clinicians from these hospitals are experienced at treating MDR-PTB patients with chemotherapeutic drugs, meantime, they will receive training in treating MDR-PTB using Chinese herbs. The researchers are experienced in project coordination.

**Table 1:** Thirty-two sentinel hospitals were recruited in the study

Provincial-level administrative division	City	Hospital name (abbreviation)
Chongqing	Chongqing	Chongqing Public Health Medical Center (CQPHMC)
Zhejiang	Lishui	Lishui Hospital of Traditional Chinese Medicine (LSTCMH)
Yunan	Kunming	Yunnan Provincial Infectious Diseases Hospital (YNIDH)
Xinjiang	Urumqi	Chest Hospital of Xinjiang Uygur Autonomous Region (XJCH)
Tianjin	Tianjin	Tianjian Haihe Hospital (TJHHH)
Sichuan	Luzhou	The Affiliated Hospital of Southwest Medical University (SMUH)
Shanghai	Shanghai	Longhua Hospital Affiliated to Shanghai University of Traditional Chinese Medicine (LHTCMH)
Shanghai	Shanghai	Shanghai Pulmonary Hospital (SHPH)
Shanghai	Shanghai	The Central Hospital of Xuhui District (XHCH)
Shanghai	Shanghai	Shanghai Public Health Clinical Center (SHPHCC)
Shanxi	Xi'an	Shaanxi Provincial Institute for Tuberculosis Control and Prevention (SXITCP)
Shanxi	Xi’an	Xian Chest Hospital (XACH)
Shangdong	Weifang	Weifang Respiratory Disease Hospital (WFRDH)
Inner Mongolia	Huhehot	The No.4 People's Hospital of Inner Mongolia Autonomous Region (FHIMAR)
Liaoning	Shenyang	Shenyang Chest Hospital (SYCH)
Jiangxi	Nanchang	Jiangxi Provincial Chest Hospital (JXCH)
Jiangsu	Zhenjiang	The No.3 People's Hospital of Zhenjiang (ZJTPH)
Jiangsu	Nanjing	The No.2 People's Hospital of Nanjing (NJSH)
Jilin	Changchun	Changchun Infectious Diseases Hospital (CCIDH)
Jilin	Changchun	Jilin Provincial Tuberculosis Hospital (JLTH)
Hunan	Changsha	The Central Hospital of Changsha (CSCH)
Hunan	Changsha	Hunan Provincial Chest Hospital (HNCH)
Hubei	Wuhan	Wuhan Chest Hospital (WHCH)
Henan	Xinxiang	The First Affiliated Hospital of Xinxiang Medical College (XXFH)
Hebei	Shijiazhuang	Hebei Provincial Chest Hospital (HBCH)
Guangdong	Shenzhen	The People's Hospital of Shenzhen (SZTH)
Guizhou	Guiyang	Guiyang Public Health Treatment Center (GYPHTC)
Fujian	Fuzhou	Fuzhou Pulmonary Hospital (FZPH)
Beijing	Beijing	Beijing Chest Hospital (BJCH)
Beijing	Beijing	The 8th Medical Center of Chinese PLA General Hospital (PLAEMC)
Anhui	Hefei	Anhui Provincial Chest Hospital (AHCH)
Anhui	Hefei	Hefei Infectious Diseases Hospital (HFIDH)

### Sample size calculation

In the present study, the module “Superiority by a Margin Tests for the Difference Between Two Proportions” in PASS 15 Power Analysis and Sample Size Software (NCSS, LLC. Kaysville, Utah, USA) was used to calculate the sample size.

#### Qi-yin deficiency patients

The following two hypotheses will be tested: H_1__0_: pA1 = pB1; H_1__1_: pA1 > pB1; where α (type I error rate) = 0.025 (one-side), 1 − β (power) = 0.9. According to the literatures [13, 16], the cure rate was 44.0% for MDR-PTB treated with bedaquiline [13]. In our previous study, the cure rate for MDR-PTB patients was 66.1% treated with short course chemotherapy and Chinese herbs [16]. Hence, if pA1 (treatment group) = 66.1%, pB1 (control group) = 44.0%, and the ratio of treatment group to control group is 2:1. The number of sample size of treatment group and control group was calculated to be 154 and 77, respectively. The follow-up failure rate is estimated to be 10%, and so the minimum number will be 86 and 172 in control group and treatment group, respectively.

#### Yin deficiency lung heat syndrome patients

The following two hypotheses will be tested: H_2__0_: pA2 = pB2; H_2__1_: pA2 > pB2; where α (type I error rate) = 0.025 (one-side), 1 − β (power) = 0.9. According to the literatures [13, 16], the cure rate was 44.0% for MDR-PTB treated with bedaquiline [13]. In our previous study, the cure rate for MDR-PTB patients was 64.2% treated with short course chemotherapy and Chinese herbs [16]. Hence, if pA2 (treatment group) = 64.2%, pB2 (control group) = 44.0%, and the ratio in the treatment to control groups is 2:1, the number of patients in the treatment and control groups was calculated to be 188 and 94, respectively. Follow-up failure rate was estimated to be 10%, and so the minimum number will be 104 and 208 in control group and treatment group, respectively.

### Diagnostic criteria

#### MDR-TB diagnostic criteria

**Fig. 2:**
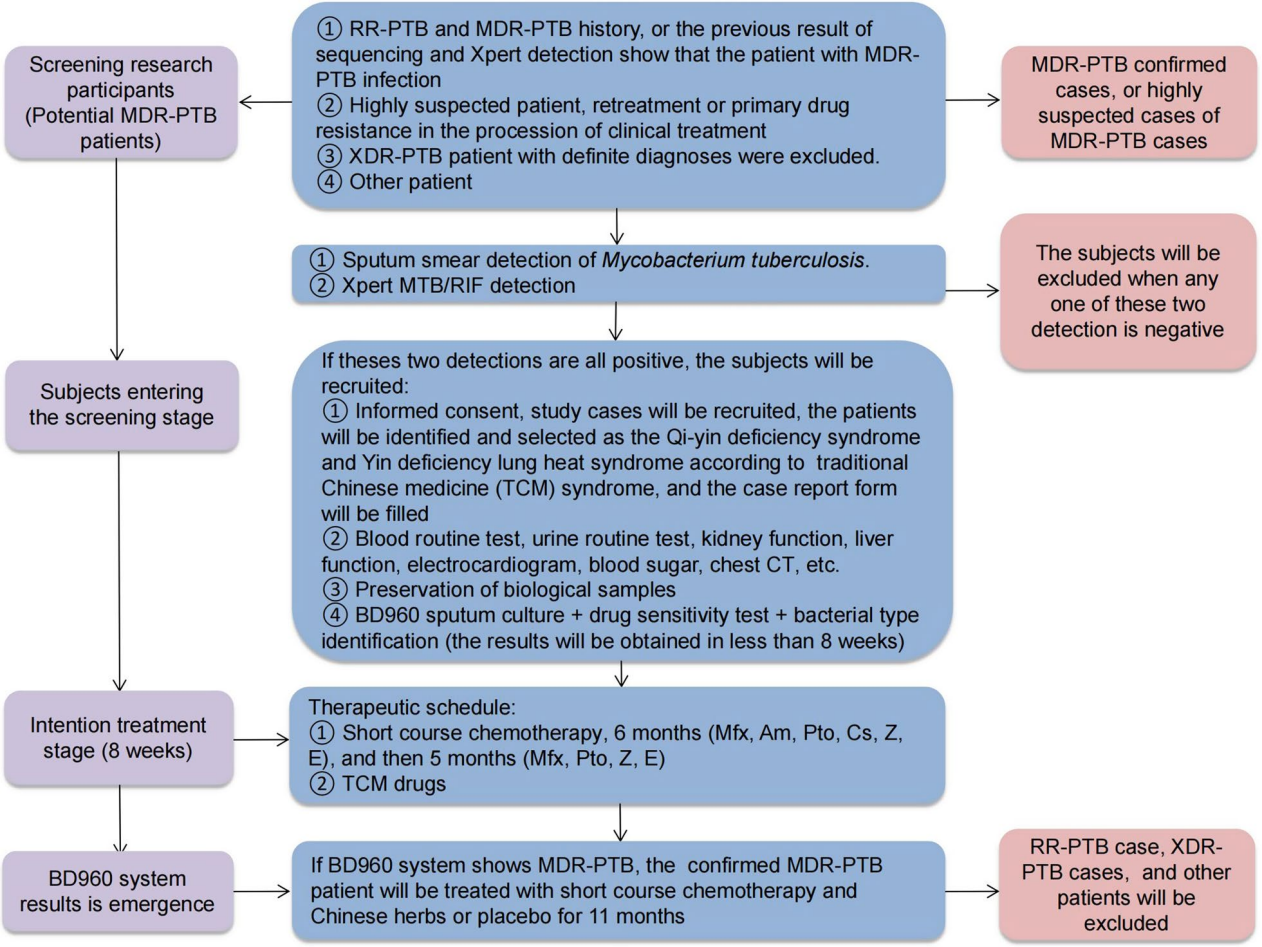
Initial screening process for patients in present study. *Am* Amikacin; *Cs* Cycloserine; *CT* Computed tomography; *E* Ethambutol (or EMB); *MDR-PTB* Multi-drug resistant pulmonary tuberculosis; *Mfx* Moxifloxacin; *Pto* Prothionamide; *RR-PTB* Rifampicin resistant pulmonary tuberculosis; *TCM* Traditional Chinese medicine; *XDR-PTB* Extensively drug-resistance pulmonary tuberculosi; *Z* Pyrazinamide (or PZA)

(i) Positive culture of *M.tb* strain in the sputum or bronchoalveolar lavage fluid from patients with PTB. (ii) Patients infected with Non-*M.tb* strain will be excluded during the process of strain identification (Fig. 2). (iii) Isolated and cultured *M.tb* strain are resistant to IHN and rifampicin (RIF).

#### Traditional Chinese medicine (TCM) syndrome differentiation diagnostic criteria

The differentiation of TCM syndrome will be based on key criteria of the principal symptoms: main symptoms, secondary symptoms, tongue fur and pulse (Additional file 1: Appendix 1). (i) Qi-yin deficiency syndrome definition: main symptoms (cough, shortness of breath, fatigue), secondary symptoms (afternoon fever, night sweat, spontaneous sweat, dry mouth, dry throat, flushed cheeks), tongue fur (red tongue, little coating), pulse (thin pulse). (ii) Yin deficiency lung heat syndrome definition: main symptoms (sputum, yellow thick sputum, or blood in the sputum, chest tightness or chest pain, five upset fever), secondary symptoms (low fever, night sweat, flushed cheeks, dry throat, thirst), tongue fur (red tongue, yellow greasy moss), pulse (thready rapid pulse).

### Inclusion criteria

Subjects will be recruited at the outpatient and inpatient departments of the sentinel hospitals. Patients who meet all the following inclusion criteria will be considered for recruitment: (1) *M.tb* strain positive diagnosed with culture from sputum or bronchoalveolar lavage fluid as identified using a BACTEC MGIT 960 automated mycobacterial detection system (Becton, Dickinson and Company, Franklin Lakes, NJ, US. BD960). (2) GeneXpert MTB/RIF (Cepheid, Caribbean Drive Sunnyvale, California, United States) positive for sputum or bronchoalveolar lavage fluid sample. (3) *M.tb* strain are simultaneous resistance to IHN and RIF. (4) Cases fulfilling the diagnostic criteria for Qi-yin deficiency syndrome or Yin deficiency lung heat syndrome. (5) Patients age between 18 and 65 years. (6) No history of allergies to drugs involved in the trial. (7) Patients must agree to the treatment voluntarily and provide signed informed consent.

### Exclusion criteria

Subjects meeting one or more of the following criteria will be excluded for the trial: (1) Subjects previously treated for MDR-TB and those having received any second line drug, including any anti-mycobacterial agent, any aminoglycoside except streptomycin (Sm), any fluoroquinolone, Pto or EMB, or Cs. (2) *M.tb* strains that is resistant to Mfx and/or Am, or drugs in the same group which cannot be modified, in accordance with the WHO classification (2016 update). (3) Suffering from the disease for more than 5 years with more than 2 retreatments. (4) Chest CT indicating lung cavity diameters is greater than 5 cm, or more than 5 cavities. (5) A history of massive hemoptysis. (6) Patients with extrapulmonary TB (bronchial TB, lymphatic TB, bone TB, or brain TB). (7) Body mass index (BMI) is less than 19. (8) Patients with respiratory failure, severe peptic ulcers, cancer, acquired immune deficiency syndrome, autoimmune disease, mental disorder, or a family history of mental disorder. (9) Disabled patients as defined by law. (10) Patients unable to receive treatment with oral medication. (11) Patients who are pregnant, preparing for pregnancy, or breastfeeding. (12) Subjects with abnormal electrocardiogram (ECG). (13) Patients with abnormal liver function, the alanine aminotransferase (ALT) or aspartate aminotransferase (AST) ≥ 2 times upper limit, patients that are HBsAg, HBeAg, or HBcAg positive. (14) Diabetic patients with poor blood glucose control (fasting blood glucose > 7 mmol/L, postprandial blood glucose > 11 mmol/L). (15) Patients unlikely to comply with the study protocol.

For patients recruited to the trial, the investigators will explain the study in detail, answer questions, ensuring that informed consent is provided to each patient and an appropriate signature is obtained prior to the start of the study. After the informed consent form (ICF) has been signed, a patient will be considered “eligible” to enter the trial, and he/she will be assigned a patient number. All additional healthcare needs of participants, or compensation for healthcare, will continue to be provided.

### Allocation to study intervention

Allocation of participants to a study group will be conducted using an interactive response technology (IRT) system. Site personnel (study coordinator or specified researcher) will be required to enter or select information including but not limited to the user's ID and password, the protocol number, and the participant number. Site personnel will then be provided with a drug assignment and randomization number. The IRT system will provide a confirmation report for each participant containing the participant number, randomization number, and the assigned study intervention. Confirmation reports will be stored at the corresponding site.

### Randomization and blinding

MDR-PTB patients with Qi-yin deficiency syndrome or Yin deficiency lung heat syndrome will be randomized independently. Randomization will be performed using a stratified-block randomization method provided by professional statisticians. Randomization will be stratified by study center using a web-based randomization service provided by ChuangDa Medical Technology Company Ltd. (Shanghai, China. https://edc.trialdata.cn/). The randomization sequence will not be released until all interventions have been assigned.

The viewing of treatment allocation prior to patient registration in the study database will not be permitted, nor will it be possible to remove a patient from the study after treatment assignment has been revealed. A statistician will label the study medication and will distribute it to each study center. Outcome data after three months will be assessed by a trained investigator blinded to treatment allocation using standardized forms and procedures. An independent trial statistician will combine data for treatment allocation with the clinical data prior to reporting to a data and safety monitoring board (DSMB).

### Unblinding

In the case of an emergency, one investigator has the sole responsibility for determining if the unblinding of a participant's study intervention assignment is warranted. Participant safety will always be the first consideration when making such a determination. If the investigator decides that unblinding is warranted, the investigator will make every effort to contact the sponsor prior to unblinding the assignment of a participant so long as any medical management would not be delayed. The sponsor will be notified within 24 h of an instance of unblinding while the date and reason will be recorded in the source documentation and case report form (CRF).

## Research content

The treatment for MDR-PTB is a complex and long-term undertaking, the content of the present study involves the following aspects.

i. Sputum will be collected and the *M.tb* strain will be determined using GeneXpert testing and culture with BD960 system, DST of *M.tb* stain will performed using the BD960 system. For each patient, *M.tb* strain identification and DST will be conducted once a week during weeks 0–12, then once per month during months 4–11 (Table 2).
ii. Each patient will undergo chest computed tomography (CT), it will evaluate lesion absorption and cavity closure (Table 2).
iii. The basic demographic information of each subject will be collected using a structured questionnaire, to include age, gender, height, weight, place of birth, residence, level of education, religion, level of income, basic disease characteristics. In addition, the structured questionnaire will be used to collect details of patient lifestyle, such as level of smoking, drinking, history of silicosis, pneumoconiosis, lung infection, contact history with MDR-PTB patients, and so on (Table 2).
iv. Clinical manifestations of each patient will be recorded, including coughing, expectoration, hemoptysis, fever (low fever), fatigue, night sweats, tightness of the chest, chest pain, dyspnea, insomnia, emaciation, loss of appetite, or other symptoms (Table 2).

**Table 2:** Flowchart for the present study

Item	Screening visit day	1st month	2nd month	3rd month	4th month	5th month	6th month	7th month	8th month	9th month	10th month	11th month
Informed consent	√											
Basic information^a^	√											
Epidemiological investigation^b^	√											
Underlying disease^c^	√											
Allocation	√											
Intervention		√	√	√	√	√	√	√	√	√	√	√
Chemotherapeutic drugs + No.1 + No.3 compound		√	√	√	√	√	√	√	√	√	√	√
Chemotherapeutic drugs + No. 1 placebo + No. 3 placebo		√	√	√	√	√	√	√	√	√	√	√
Chemotherapeutic drugs + No. 2 + No. 4 compound		√	√	√	√	√	√	√	√	√	√	√
Chemotherapeutic drugs + No. 2 placebo + No. 4 placebo		√	√	√	√	√	√	√	√	√	√	√
Outcome measure												
Cure rate												√
Time to sputum culture conversion												√
Lesion absorption rate	√			√			√					√
Cavity closure rate	√			√			√					√
The effect rate of TCM syndrome	√			√			√					√
Clinical manifestations^a^	√	√	√	√	√	√	√	√	√		√	√
Chest CT	√			√			√					√
Culture of *M.tb*^a,b^	√	√	√	√	√	√	√	√	√	√	√	√
Acid-Fast Bacilli sputum smear^d,e^	√	√	√	√	√	√	√	√	√	√	√	√
DST^d,e^	√	√	√	√	√	√	√	√	√	√	√	√
Safety assessments												
Physical examination^d^	√	√	√	√	√	√	√	√	√	√	√	√
Vital signs^d,f^	√	√	√	√	√	√	√	√	√	√	√	√
Physical examination^d^	√	√	√	√	√	√	√	√	√	√	√	√
Complete ophthalmological examination^d^	√	√	√	√	√	√	√	√	√	√	√	√
Blood routine^d^	√	√	√	√	√	√	√	√	√	√	√	√
Urine routine^d^	√	√	√	√	√	√	√	√	√	√	√	√
Liver function^d^	√	√	√	√	√	√	√	√	√	√	√	√
Kidney function^d^	√	√	√	√	√	√	√	√	√	√	√	√
Electrocardiogram^d^	√	√		√								√
Blood glucose	√	√		√								√
Adverse events recorded^d,g^	√	√	√	√	√	√	√	√	√	√	√	√

*CT* computed tomography; *DST* drug susceptibility testing; *M.tb*: *Mycobacterium tuberculosis*; *TCM* traditional Chinese medicine

^a^Basic information includes age, gender, height, weight, occupation, education, religion, income, residence, etc.

^b^Epidemiological information includes: smoking, drinking, floating population, vagrant, use of corticosteroids, use of immunosuppressive agents, working within dusty, high-temperature or high-humidity environments, presence of silicosis, pneumoconiosis, pulmonary infection, traveling long distances, contact with TB or MDR-PTB patients, long term use of antibiotics, etc.

^c^Underlying diseases include: AIDS, hepatitis B, chronic obstructive pulmonary disease, asthma, diabetes, nervous system disease, hyperthyroidism, hypothyroidism, hypertension, hyperlipidemia, hyperuricemia, cardiovascular disease, autonomic dysfunction, hyperlipidemia, pulmonary malignancy, etc.

^d^These examinations or experiments will be conducted in each subject on the day of screening and each week during months 1–3

^e^After the 11-month treatment, *M.tb* identification and DST will be performed monthly from months 12–16. The aim of the study is to assess whether MDR-PTB patients are cured by the end of month 11

^f^Vital signs include pulse rate, blood pressure, and respiratory rate

^g^Adverse events include: systemic reactions (allergic reaction, headache, fever, fatigue), respiratory system (upper respiratory tract infection, spontaneous, lung infection, lung inflammation), digestive system (vomiting, impaired liver function, nausea, bloating, gastrointestinal discomfort, constipation, diarrhea, oral discomfort, dysphagia), skin system (mucocutaneous reactions, induration, erythema, edema, rash, pruritus), neurological system (neuro-cerebellar reactions, psychiatric disorder, reduced muscle strength, paresthesia). Musculoskeletal reactions (arthralgia, arthritis, myalgia). Laboratory test results (elevated erythrocytes, reduced hemoglobin, elevated hemoglobin, reduced leukocytes, elevated leukocytes, elevated platelets, reduced platelets, elevated uric acid, elevated urinary red blood cells, urine leukocytosis, elevated urinary protein, elevated urine, elevated alanine aminotransferase, elevated aspartate aminotransferase, reduced total bilirubin, elevated total bilirubin, reduced blood urea nitrogen, elevated blood urea nitrogen, reduced creatinine, elevated creatinine)

### Investigational drug

#### Therapeutic schedule

The short course chemotherapy regimen for MDR-PTB treatment will be consisted of a 6-month intensive treatment period (6MfxAmPtoCsZE) and a 5-month onsolidation phase (5MfxPtoZE). These chemotherapeutic drugs prescribed for each subject for the entire trial. In addition, the MDR-PTB cases with Qi-yin deficiency will be allocated to the treatment or control group at a ratio of 2:1 (treatment group:control group) using a randomization process. The treatment groups will receive chemotherapeutic drugs plus Chinese herbs granules (1 + 3 granules), whereas the control group will receive chemotherapeutic drugs plus Chinese herbs placebo (1 + 3 placebo granules). Meanwhile, MDR-PTB patients with Yin deficiency lung heat syndrome will be allocated randomly to the a treatment or control group at a ratio of 2:1 (treatment group: control group). The treatment group will be treated with chemotherapeutic drugs plus Chinese herbs granules (2 + 4 granules), and the control group will be treated with chemotherapeutic drugs plus Chinese herbs placebo (2 + 4 placebo granules, Table 3).

**Table 3:** Drugs prescribed for MDR-PTB cases in present study

Drug	Dosage form	Treatment per day	
		Weight < 50 kg	Weight ≥ 50 kg
Modern chemotherapeutic drugs			
Amikacin (Am)	Injection	0.40 g (once a day)	0.60 g (once a day)
Moxifloxacin (Mfx)	Tablet	0.40 g (once a day)	0.40 g (once a day)
Cycloserine (Cs)	Capsule	0.25 g (twice a day)	0.25 g (twice a day)
Promethionine (Pto)	Tablet	0.30 g (twice a day)	0.40 g (twice a day)
Pyrazinamide (Z)	Tablet	1.50 g (once a day)	1.50 g (once a day)
Ethambutol (E)	Tablet	0.25 g (twice a day)	0.25 g (three times a day)
TCM compound			
No.1 compound	Granules	8.20 g (twice a day)	8.20 g (twice a day)
No.2 compound	Granules	6.92 g (twice a day)	6.92 g (twice a day)
No.3 compound	Granules	6.60 g (twice a day)	6.60 g (twice a day)
No.4 compound	Granules	6.47 g (twice a day)	6.47 g (twice a day)
No.1 placebo	Granules	8.20 g (twice a day)	8.20 g (twice a day)
No.2 placebo	Granules	6.92 g (twice a day)	6.92 g (twice a day)
No.3 placebo	Granules	6.60 g (twice a day)	6.60 g (twice a day)
No.4 placebo	Granules	6.47 g (twice a day)	6.47 g (twice a day)

No.1 TCM herb granule 1 will be will be formulated as mixed granules for single TCM herb administration and packed in food-grade plastic bags (8.20 g/bag), the granules will comprise extracts of *Radix Astragali (Huang Qi)* 20 portions, *Rhizoma Curculiginis (Xian Mao)* 4 portions, *Fructus Rubi (Fu Pen Zi)* 2 portions, *Fructus Ligustri Lucidi (Nv Zhen Zi)* 10 portions, *Radix Rehmanniae (Di Huang)* 10 portions, etc.

No.2 TCM herb granule 2 will be will be formulated as mixed granules for single TCM herb administration and packed in food-grade plastic bags (6.92 g/bag), the granules will comprise extracts of *Rhizoma Polygonati (Huang Jing)* 6 portions, *Rhizoma Atractylodis Macrocephalae (Bai Zhu)* 5 portions, *Radix Arnebiae (Zi Cao)* 3 portions, etc.

No.3 TCM herb granule 3 will be will be formulated as mixed granules for single TCM herb administration and packed in food-grade plastic bags (6.6 g/bag), the granules will comprise extracts of *Rhizoma Imperatae (Bai Mao Gen)* 15 portions, *Gypsum Fibrosum (Shi Gao)* 10 portions, *Radix Arnebiae (Zi Cao)* 6 portions, *Herba Hedyoti Diffusae (Bai Hua She She Cao)* 20 portions, etc.

No.4 TCM herb granule 4 will be will be formulated as mixed granules for single TCM herb administration and packed in food-grade plastic bags (6.47 g/bag), the granules will comprise extracts of *Radix Stemonae (Bai Bu)* 6 portions, *Spica Prunellae (Xia Ku Cao)* 5 portions, *Herba Violae (Zi Hua Di Ding)* 5 portions, etc.

TCM herb placebo (No.1 placebo, No.2 placebo, No.3 placebo and No.4 placebo) will be formulated using supplementary materials (starch, lactose, microcrystalline cellulose, talcum powder, caramel, bitter compounds, etc.), and it will be packed in food-grade plastic bags (8.20 g/bag, 6.92 g/bag, 6.60 g/bag, 6.47 g/bag, respectively)

*MDR-PTB* Multidrug-resistant plumonary tuberculosis; *TCM* traditional Chinese medicine

#### Dispensing medication

A drug administrator at each center will have the responsibility for the storage, distribution, recovery, record keeping, and retrieval of the experimental drugs. The chemotherapeutic drugs + Chinese herbs or chemotherapeutic drugs + Chinese herbs placebo will be provided to each subject by enrollment sequence. The grouping allocation of patients will remain unchanged throughout the trial. Drugs will be distributed at each follow-up examination during this study, while any unused drugs will be recovered during subsequent drug allocation.

#### Drugs storage

All supplies of the chemical synthetic drugs and Chinese herbs will be stored at room temperature and protected from light, in a room with limited access, or within a locked cabinet in appropriate environmental conditions. Access to the study medication will be restricted to designated trial personnel. All medication will remain in the original packaging as delivered by the drug suppliers. The storage conditions and expiry date will be supplied with the investigational materials. A monitor will periodically check all supplies of study medication held by each investigator for accountability, and to ensure appropriate conditions of storage of the medication are utilized. At the end of the trial, all unused study medication will be collected by the monitor and returned to the sponsor, unless other arrangements are agreed.

### Endpoints

#### Efficacy endpoints

As shown in Table 4, the primary efficacy endpoint is the cure rate, and the secondary efficacy endpoints are time to sputum culture conversion, lesion absorption rate, cavity closure rate and the effect rate of TCM syndrome between treatment group provide with chemotherapeutic drugs + Chinese herbs and control group treated with chemotherapeutic drugs + Chinese herbs placebo in Qi-yin deficiency patients, or in Yin deficiency lung heat syndrome.

**Table 4:** Measurement endpoints in present study

Item	Definition or description
Primary endpoint	Cure rate is defined as will be five consecutive negative liquid cultures from sputum samples at a time interval of 30 days at the end of treatment
Secondary endpoints	Time to sputum culture conversion will be calculated as the interval in days between the date of treatment initiation for MDR-PTB and the date of the first of the 2 consecutive negative sputum cultures from sputa collected at least 30 days apart
	Cavity change rate (%) = 100% × (number of cavity change patients/number of patients). For changes in cavity size, scar healing, and block healing or disappearance are defined as closed. A reduction in cavity size by 1/2 or more is defined as reduced, a reduction in cavity diameter by less than 1/2 is classified as unchanged, and increased cavity diameter by 1/2 or more is defined as increased. CT results were assessed and interpreted independently by two experienced radiologists who were blinded to the study design. If inconsistent, a third expert will be invited to re-read
	Lesion absorption rate (%) = 100% × (number of lesion absorption subjects/number of subjects). The clinical criteria for CT changes are as follows [15], absorption of 1/2 or more of the lesions will be classified as significant absorption, while absorption of less than 1/2 of the lesions or an increase or emergence of new lesions will be classified as deterioration
	Effect rate of the TCM syndrome = 100% × (clinically cured cases + cases with a notable effect + cases effectively treated)/total number of cases. The efficacy was demonstrated by four possible descriptors in accordance with the TCM clinical research principles [17], namely clinical cure, notable effect, effective or ineffective. The specific classification criteria are as follows: I) clinical cure: rate of reduction in TCM syndrome scores ≥ 95%; II) notable effect: rate of reduction in TCM syndrome scores between 70 and 95%; III) effective: reduction in TCM syndrome score between 30 and 70%; IV) ineffective: reduction in TCM syndrome scores < 30%
Safety endpoints	
Adverse events	
Systemic reaction	Allergic reaction, headache, fever, fatigue
Laboratory tests	Elevated erythrocytes, reduced hemoglobin, elevated hemoglobin, reduced leukocytes, elevated leukocytes, elevated platelets, reduced platelets, elevated uric acid, etc
Respiratory system	Upper respiratory tract infection, spontaneous lung infection, lung inflammation
Digestive system	Vomiting, impaired liver function, nausea, bloating, gastrointestinal discomfort, constipation, diarrhea, oral discomfort, dysphagia
Skin system	Mucocutaneous, induration, erythema, edema, rash, pruritus
Neurological system	Neuro-cerebellar or psychiatric reactions, Muscle strength reduction, Paresthesia (burning, tingling), Neuro-sensory reaction
Musculoskeletal	Arthralgia (joint pain), arthritis, myalgia
Serious adverse events	Death, life-threatening reaction requiring inpatient hospitalization or prolongation of existing inpatient hospitalization, congenital abnormality or birth defect, persistent or significant disability or incapacity, or otherwise considered medically significant by the investigator

*CT* computed tomography; *TCM* Traditional Chinese medicine

#### Safety endpoints

Safety will be separately evaluated in terms of AEs (Table 4), clinical laboratory tests, ECG, physical examinations, and vital signs (Additional file 1: Appendix 1) for each trial period (screening stage, therapy process, and follow-up period).

AE is any inappropriate medical occurrence in a patient administered a pharmaceutical product that is not necessarily causally associated with the treatment. An AE can represent any unfavorable or unintended manifestation (including abnormal laboratory finding), symptoms, or disease temporally associated with the use of an investigational medicinal product, whether or not it is related to its administration. All AEs will be followed until they have abated, or until a stable situation has been reached. Depending on the event, follow-up may require additional tests or medical procedures, and/or referral to a general physician or a medical specialist.

Serious adverse event (SAE) is any unfavorable medical occurrence that shows in Table 4. SAEs need to be reported by the end of the study in China, as defined in the protocol.

Serious adverse reaction (SAR) is judged by either the reporting investigator or the sponsor as having a reasonable causal relationship. The expression “reasonable causal relationship” in general is used to indicate that there is evidence or an argument to suggest a causal relationship. Factors to consider when assessing causality of SARs are: (i) the nature of the reaction, (ii) timing of the reaction, and (iii) its relationship to the dose (Additional file 1: Appendix 1).

### Discontinuation of study intervention

Participation in the clinical trial will be voluntary, all subjects having the right to suspend their consent from the trial prematurely, at any time and without stating a reason, and without disadvantaging any potential future medical treatment. In rare instances, it may be necessary for a participant to permanently discontinue study intervention (definitive discontinuation or withdrawal). Reasons for definitive discontinuation of the study intervention may include participant request, investigator request, pregnancy, protocol deviation (no longer satisfying all inclusion criteria, or fulfilling one or more of the exclusion criteria).

Note that discontinuation of study intervention does not represent a withdrawal from the study. If trial intervention is definitively discontinued, the participant will remain in the study and be evaluated for safety, immunogenicity, and potential efficacy. Follow-up for any evaluation required at the time of discontinuation will be conducted.

Discontinuation of study intervention, it must be documented on the appropriate CRF and in the medical records, including the participant has discontinued from further administration of study intervention alone, or also from the study procedures, post-treatment study follow-up, and/or future collection of additional information.

### Participant withdrawal from the study

A participant may withdraw from this trial at any time at his/her own request. Reasons for discontinuation from the study may include the following: (1) Refusal to attend additional follow-up examinations. (2) Lost to follow-up. (3) Death. (4) Advice to withdraw because of poor compliance, comorbidities, or serious adverse events. (5) Participant request. (6) Investigator request. (7) Protocol deviation. (8) Patients who withdraw on their own due to poor curative effect, adverse reactions, or other reasons. (9) After allocation to a particular group, it is determined that some other drugs are required for treatment.

Participants should notify the investigator in writing of their decision to withdraw consent from future follow-up at the earliest opportunity. Withdrawal of consent should be explained in detail in the medical records by the investigator, and whether withdrawal is only from further receipt of study intervention or also from study procedures and/or post-treatment study follow-up, to be entered on the appropriate page of the CRF.

### Lost to follow-up

A participant will be considered lost to follow-up if he or she repeatedly fails to return for scheduled visits and cannot be contacted by the study site. The following actions will be taken if a participant fails to attend a required study visit. The site will attempt to contact the participant and reschedule the missed visit as soon as possible and counsel the participant on the importance of maintaining the assigned visit schedule and ascertain whether or not the participant wishes to and/or should continue in the study.

Prior to a participant being deemed lost to follow-up, the investigator or researcher will make every effort to regain contact with the participant (if possible). The site coordinator will make at least 3 telephone calls and, if necessary, a certified letter to the participant's last known mailing address, or a locally equivalent method, to prevent loss to follow-up. These contact attempts will be documented in the participant's medical records.

If the participant continues to be unreachable, he/she will be considered to have withdrawn from the study.

### Criteria for subjects’ rejection

Subjects fulfilling one or more of the following criteria will be rejected for the trial and the reasons will be recorded in detail. Their CRF will be maintained for examination purposes: (1) Cases not conforming to the inclusion criteria. (2) Cases conforming to the exclusion criteria. (3) Cases have received the course of standard chemotherapy regimen for less than 3 months. (4) Cases without any recorded test results. (5) Actual drug was not within the range of the planned dose (80–120%), although the study was completed. (6) Cases in which efficacy cannot be evaluated due to the use of a prohibited drug. 7) Cases in which the course of standard chemotherapy was less than 3 months. (8) Cases consume Chinese herbs during the trial.

### Collection of samples

#### Sputum

Sputum will be collected once per week during weeks 0–12, and once per month during months 4–11. When collecting sputum, patients will be asked to spit out any water in their mouth and take a deep breath prior to coughing up sputum into the collection vessel. Sputum will be induced prior to collection by asking patients to rinse their mouth with water, and then using an ultrasonic atomizer to spray 7 ml of 3% hypertonic saline over 15 min after which patients will attempt to cough up sputum from deep within their lungs. Qualified sputum specimens should be purulent and cheese or mucus-like, preferably with a volume of 3–5 ml. The container for collecting the sputum specimens will be an international, universal screw cap sputum container marked with the patient's name, identification number, inspection items, sputum specimen serial number, and date of production. The sputum will be submitted for inspection within 24 h.

#### Venous blood and urine

Venous blood and morning urine sample will be collected at the screening visit time, once a week during weeks 0–12, then once a month during months 4–11. A proportion of them will be analyzed by routine laboratory testing and the remainder frozen at − 80 °C for future study.

### Laboratory detection

#### Ziehl–Neelsen method to find M.tb strain

Smears for *M.tb* strain will be performed by Ziehl–Neelsen staining using an Acid-Fast Stain Kit (Cat. G1170. Beijing Solarbio Science & Technology Co., Ltd, Beijing, China). *M.tb* strain (H37Rv, ATCC 27294; or H37Ra, ATCC 21577) and *Escherichia coli* (ATCC25922) will be used as quality control.

#### M.tb strain culture and DST

The sputum specimens from each subject will be digested and decontaminated using an N-acetyl-l-cysteine-sodium hydroxide method. DST will be performed at the clinical laboratories. The final concentration of each drug in the culture medium will be defined by the *M.tb* strain growth indicator tube operating procedure guidelines provided by Becton Dickinson and Company.

#### ECG

At the specified time points, ECGs (supine, following at least 5 min rest) will be recorded by an ECG provider. All ECGs will be reviewed by a cardiologist.

#### Blood samples

Samples will be collected to measure the prothrombin time, hematocrit, hemoglobin levels, and platelet, red blood cell (RBC), white blood cell (WBC), and differential WBC counts (neutrophils, lymphocytes, monocytes, eosinophils, basophils). In addition, total protein, alkaline phosphatase (ALP), AST, ALT, lactate dehydrogenase (LDH), total cholesterol, triglycerides, direct bilirubin, indirect bilirubin, total bilirubin, blood urea nitrogen (BUN), uric acid, creatinine phosphokinase (CPK), cardiac troponin I, electrolytes (sodium, potassium, phosphate, chloride, and chloride, calcium), glucose, pancreatic amylase, lipase, human serum albumin, and trypsin-like immunoreactivity will be assessed. Gastrin and pepsinogen will also be measured for gastrointestinal evaluation (Additional file 1: Appendix 1).

#### Urine samples

A midstream urine sample will be provided for levels of protein, glucose, occult blood, ketones, bilirubin, urobilinogen, nitrite, and specific gravity, the results of which will be documented in the source documents. If abnormal, microscopic examination for WBCs, RBCs, epithelial cells, crystals, bacteria, or casts will be conducted.

### Data management and monitoring

#### Data safety monitoring board (DSMB)

The trial will be monitored by an independent DSMB to ensure data safety, and an independent data monitoring committee (DMC) will also be established for this trial. DMC will include at least one statistician, one TB expert and one methodology specialist. DMC will meet annually to review all collected data and may meet more frequently if required after analysis of the available data. The DMC will advise the trial management committee and the independent trial steering committee on the safety of the trial. All unexpected SAEs will be reported to the trial management committee and the trial sponsors by facsimile within 24 h of their occurrence, or learning of the occurrence, by local investigators. All data regarding AEs will be made available to the DMC for review.

#### Electronic data management system

An electronic database will be established to manage trial data. (1) Design electronic case report form (eCRF): the data manager will construct an eCRF specific for this research project and medical records. (2) Authority allocation: the sponsor, monitors and inspectors, the data manager will create accounts and grant the appropriate permissions to access the electronic clinical data management system (eCDM). In instance, researchers in each center will only see content appropriate for that center and only have the right to modify the data, whereas the sponsors will be limited to viewing from all centers. The monitors and inspectors will be able to read the case histories without having permission to modify the data, but they can add comments or questions. (3) Data entry: Clinical investigators or data entry officers designated by the investigator will input the data from the study records into eCRFs in a timely and accurate manner. (4) Data query and questions: Monitors will inspect data using the eCDM. If errors are evident, they can raise queries online at any time, to which researchers will provide answers online, and modify any incorrect data. (5) Data locking and exporting: After each subject has completed particular tests and the data has been reviewed by the monitor, the data manager will lock data. The data manager will import the study data into a designated database after all data has been entered, then provide it to statisticians for data analysis.

### Statistical analysis

Full analysis set (FAS): Data consisting of all eligible cases. Baseline data and demographic characteristics will be compared. Where the main efficacy indicators are absent, previous results will be carried forward according to intention to treat (ITT) analysis. Missing values of the secondary efficacy indicators will not be date-carried-forward or compared, but analyzed using data actually present within the FAS.

Per protocol set (PPS): The set of cases satisfying the inclusion criteria, not excluded by the exclusion criteria, and completed the treatment regimen, that is, the analysis of cases that correctly has undergone the test regimen with good compliance, and completed all CRF requirements (PP analysis). Per protocol analysis will be used mainly for principal efficacy indicators.

Security data set (SS): This represents patients that have received at least one treatment, with actual data on security indicators recorded. Missing security values will not be carried forward. The incidence of adverse reactions within a SS represents the denominator for case numbers.

The analysis will encompass all randomized patients using ITT, except for those who fail to respond to prednisolone following randomization. Exclusion of these patients will not result in bias as: (1) these dropouts will have occurred prior to commencement of randomized treatment, and (2) clinicians will be unaware of the treatment assigned to each patient. It is anticipated that rates of missing data will be low, and there will be no need for imputation.

For the baseline data of MDR-PTB cases with Qi-yin deficiency syndrome, the continuous values (age, height, weight, BMI) will be compared across the treatment and control groups using a student's *t*-test (*t* value) or a Wilcoxon rank-sum test (Z value). Categorical values (gender, occupation, contacting MDR-PTB patients, etc.) will be compared between two groups using Chi-square test (χ^2^ value). Meantime, the same analysis process and method will be performed between two groups in MDR-TB patients with Yin deficiency lung heat syndrome.

For the primary efficacy endpoint, the rate difference (RD) for cure rate between chemotherapeutic drugs + Chinese herbs group and chemotherapeutic drugs + Chinese herbs placebo group will be calculated in MDR-PTB cases with Qi-yin deficiency syndrome. Meantime, the point estimation values and 95% confidence intervals (*CIs*) of RD will be reported. In addition, the same analysis process and method will be conducted between two groups in MDR-PTB patients with Yin deficiency lung heat syndrome.

For the secondary efficacy endpoints, the RDs for lesion absorption rate, cavity change rate and effect rate of the TCM syndrome between two groups will be calculated in MDR-PTB cases with Qi-yin deficiency syndrome, with which the point estimation values and 95% *CI*s values will be reported. Meantime, the median difference of time to sputum culture conversion between two groups will be compared using Kaplan–Meier method (Log-rank, Breslow, Tarone tests). In addition, the same analysis process and method will be carried out between two groups in MDR-PTB patients with Yin deficiency lung heat syndrome.

For the safety endpoints, which are all binary variables (abnormal liver function, electrocardiographic abnormality, abnormal creatinine, etc., Additional file 1: Appendix 1), statistical description (percentage and rate) will be conducted for the MDR-PTB patients with Qi-yin deficiency syndrome and Yin deficiency lung heat syndrome, respectively.

## Discussion

Over recent years, only gentle decline in the TB prevalence has been experienced in China [18–20], in part because of the MDR-PTB emergence and prevalence [21, 22]. For both individual patients with MDR-PTB and national TB programs, a shorter duration of treatment that is effective and beneficial [23]; and standardized treatment using chemical synthesized drugs are crucial for treating MDR-PTB [24, 25]. Although it represents too great a financial pressure for patients who may be prone to being affected by poverty as a result [21, 26]. In addition, many of patients abandon treatment because of long-course treatment and the substantial side-effects. This reduces the cure rate and enhances the infectious risk for other individuals.

Chinese herbs have a long history for treating TB patients, it has been popular in regions where health resources are limited, including county hospitals or clinics in townships or rural areas [27]. Chinese herbs are considered to be able to treat MDR-PTB because they may improve an individual’s natural immune system and ameliorate clinical manifestations. Therefore, it is believed that the combination of Chinese herbs with chemotherapeutic drugs for treating MDR-PTB may compensate for the deficiencies in chemical synthetic drugs used alone. Consequently, it is somewhat urgent that Chinese herbs compound preparations will be developed and industrialized to treat MDR-PTB.

Although Chinese herbs have been used to treat MDR-PTB in the real world, high quality clinical evidence of MDR-PTB treated with Chinese herbs have not yet been collected. Thus, the large-scale RCT was designed to objectively investigate the roles of Chinese herbs for treating MDR-PTB with chemical synthetic drugs. It will provide objective knowledge that Chinese herbs can or cannot improve the therapeutic effect of chemical synthetic drugs.

In fact, this study is one loading test. It involves chemotherapeutic drugs and Chinese herbs. If there is a statistical difference between chemotherapeutic drugs + Chinese herbs group compared with chemotherapeutic drugs + Chinese herbs placebo, and the clinical efficacy of chemotherapeutic drugs + Chinese herbs group is high, it can only show that Chinese herbal can improve the clinical efficacy of chemotherapeutic drugs. It cannot conclude that Chinese herbal can effectively treat MDR-PTB. It is that synergistic effect may be existence between chemotherapeutic drugs and Chinese herbs. However, no adopt appropriate research was conducted to explore the possible interaction effect (synergistic effect, antagonistic effect) in study population. As a matter of fact, the interaction between chemotherapeutic drugs and Chinese herbs should be discover by mechanism research and animal experiments.

Chinese herbal contains a large number of components. It is necessary to discover the components and monomers before starting clinical study, and the blood concentration and metabolism produce of Chinese herbal components also need be discover before the previous research. In addition, it also requires strict quality control to ensure the identical components and concentrations in different batches of Chinese herbs, it can increase the efficacy comparability and it is very crucial to the present study.

In conclusion, the present study will provide objective evidence to evaluate the clinical efficacy of Chinese herbs for improving curative effect of chemical synthesized drugs.

## Supplementary Information

**Additional file 1: Table S1.** Chemotherapeutic drugs are used for treating MDR-TB patients stated by WHO guidelines. **Table S2.** The basic concept of drug resistance for *Mycobacterium tuberculosis*. **Table S3.** Drugs prescribed for MDR-PTB cases in these present study. **Table S4.** Evaluation criteria of traditional Chinese medicine syndrome score in MDR-PTB. **Table S5.** The follow-up process and time point for each subject in the study. 

## Data Availability

All relevant data will be available after completion of the research, ensuring participant confidentiality.

## Abbreviations

AEs: Adverse events
ALP: Alkaline phosphatase
ALT: Alanine aminotransferase
Am: Amikacin
Amx: Amoxicillin
AST: Aspartate aminotransferase
BD960: BACTEC MGIT 960 system
Bdp: Bedaquinoline
BMI: Body mass index
BP: Blood pressure
BUN: Blood urea nitrogen
Cfz: Clofazimine
Cln: Cilastatin
*CI*: Confidence interval
Clv: Clavulanic acid
Cm: Capreomycin
Cs: Cycloserine
CPK: Creatine phosphohykinase
CRF: Case report form
CT: Computed tomography
DMC: Data Monitoring Committee
DOTS: Directly observed treatment short
DR-TB: Drug resistant tuberculosis
DSMB: Data and Safety Monitoring Board
DST: Drug susceptibility testing
ECG: Electrocardiogram
eCRF: Electronic case report form
EMB: Ethambutol
ETH: Ethionamide
Eto: Ethionamide
FAS: Full analysis set
Gfx: Gatifloxacin
ICF: Informed consent form
INH: Isoniazid
IP: Investigational product
Ipm: Imipenem
IRT: Interactive response technology
ITT: Intention to treat
Km: Kanamycin
LDH: Lactate dehydrogenase
Lfx: Levofloxacin
Lzd: Linezolid
MDR-TB: Multidrug-resistant tuberculosis
MDR-PTB: Multidrug-resistant plumonary tuberculosis
Mfx: Moxifloxacin
Mpm: Meropenem
*M.tb*: *Mycobacterium tuberculosis*
RR: Rate ratio
PAS: Paza-aminosalicylate
PPS: Per protocol set
PTB: Plumonary tuberculosis
Pto: Prothionamide
PZA: Pyrazinamide
RBC: Red blood cell
RCT: Randomized controlled trial
RD: Rate difference
RIF: Rifampicin
RR-TB: Rifampicin-resistant tuberculosis
SAEs: Serious adverse events
SARs: Serious adverse reactions
Sm: Streptomycin
SS: Security data set
TB: Tuberculosis
TCM: Traditional Chinese Medicine
Trd: Terizidone
WBC: White blood cell
WHO: World Health Organization
XDR: Extensively drug resistant
