# Supplementary material for: Efficacy of integrating short-course chemotherapy with Chinese herbs to treat multi-drug resistant pulmonary tuberculosis in China: a study protocol
Source: Infect Dis Poverty. 2021 Nov 6;10:131. doi: 10.1186/s40249-021-00913-5 (PMC8572065; doi:10.1186/s40249-021-00913-5)
Supplement: Supplementary file 1 — Additional file 1: Table S1. Chemotherapeutic drugs are used for treating MDR-TB patients stated by WHO guidelines. Table S2. The basic concept of drug resistance for Mycobacterium tuberculosis. Table S3. Drugs prescribed for MDR-PTB cases in these present study. Table S4. Evaluation criteria of traditional Chinese medicine syndrome score in MDR-PTB. Table S5. The follow-up process and time point for each subject in the study. [file 40249_2021_913_MOESM1_ESM.doc]

**Supplementary Appendix 1**

**Part 1**

Table 1 Chemotherapeutic drugs are used for treating MDR-TB patients stated by WHO guidelines

| Edition | Group | Chemotherapeutic drugs |
| --- | --- | --- |
| 2016 guidelines | A | Levofloxacin (Lfx), Moxifloxacin (Mfx), Gatifloxacin (Gfx) |
|  | B | Amikacin (Am), Capreomycin (Cm), Kanamycin (Km), Streptomycin(Sm) (all are injections) |
|  | C | Ethionamide (Eto) or Prothionamide (Pto), Cycloserine (Cs) or terizidone (Trd), Linezolid (Lzd), Clofazimine (Cfz) |
|  | D1 | Pyrazinamide (PZA), Ethambutol (EMB) |
|  | D2 | Dramani (Dim), Bedaquinoline (Bdp) |
|  | D3 | P-aminosalicylic acid (PAS), Amoxicillin-clavulanic acid (Amx-Clv) |
|  |  | Meropenem (Mpm), Imipenem-cilastatin (Ipm-Cln) |
| 2018 guidelines | Group^1^ | Levofloxacin (Lfx), Moxifloxacin (Mfx), Gatifloxacin (Gfx) |
|  |  | Bedaquinoline (Bdp), Linezolid (Lzd) |
|  | Group^2^ | Clofazimine (Cfz), Cycloserine (Cs) or Terizidone (Trd) |
|  | Group^3^ | Pyrazinamide (PZA, Z), Ethambutol (EMB), Dramani (Dim) |
|  |  | Imipenem-cilastatin (Ipm-Cln, or Meropenem, Mpm) |
|  |  | Amikacin (Am, or Streptomycin, Sm), Ethionamide (Eto) |

Notes: 1.The regimen should consist of all three types of drug unless this is unsuitable. 2. Two drugs are added at the same time unless this is unsuitable. 3. If drugs from groups A and B cannot be used, add drug from this group.

Table 2 The basic concept of drug resistance for *Mycobacterium tuberculosis*

| Noun | Concept |
| --- | --- |
| Primary drug resistance | *Mycobacterium tuberculosis*（*M.tb*）strain from TB patients who have never been treated with anti-TB drugs is resistant to one or more first-line anti-TB drugs. It includes not only the natural drug resistance caused by the gene mutation of the resistant TB strains, but also the sensitive TB strain who evade the clearance of the immune system in vivo. |
| Acquired drug resistance | *M.tb* strain from TB patients is sensitive to all first-line anti-TB drugs at the beginning of treatment TB patients. However, the *M.tb* strain has gradually developed into resistance to one or more first-line anti-TB drugs in the process of treatment.Due to improper treatment and other factors, the original sensitive TB strain was killed, but a litter resistance strain was produced with mutants, and then it became the dominant strain gradually. |
| Initial drug resistance | *M.tb* strain from TB patients is resistant to one or more anti-TB drugs, but the treatment history of patient is unknown. It includes primary drug resistance and a part of acquired drug resistance that has not been confirmed. |
| Natural drug resistance | *M.tb* strain from TB patients is resistance one or more first-line anti-TB drugs before the patients treatment with first-line anti-TB drugs, that is, the TB cases infected with strain which is resistance to one or more first-line anti-TB drugs. |
| MR-TB | *M.tb* strain from TB patients is resistance only one first-line anti-TB drugs in DST in vitro. |
| PDR-TB | *M.tb* strain from TB patients is confirmed to be resistant to more than 1 first-line anti-TB drug in DST in vitro (does not including to strain which is resistance to both INH and RFP). |
| MDR-TB | *M.tb* strain from TB patients is resistance to resistance to at least both INH and REP in DST in vitro. |
| Pre-XDR-TB | *M.tb* strain from TB patients has been confirmed MDR-TB in DST in vitro, in addition, the strain is resistance one kind fluoroquinolone drug or one of second-line anti-TB drugs(three kind injectable drugs, kanamycin, amikacin, capreomycin). |
| XDR-TB | *M.tb* strain from TB patients has been confirmed MRD-TB in DST in vitro, in addition, the strain is resistane one kind  fluoroquinolone drug and one of second-line anti-TB durg (three kind injectable drugs, kanamycin, amikacin, capreomycin). |
| RFP-TB | *M.tb* strain from TB patients has been confirmed to resistance to REP in DST in vitro. |

Notes: The classifications and definitions are suitable for all patients with primary and secondary TB, including PTB and extrapulmonary TB, this classification have great significance for the TB clinical diagnosis, prevention and control.

Table 3 Drugs prescribed for MDR-PTB cases in thise present study

| Drugs | Dosage form | Treatment per day | | Manufacturer |
| --- | --- | --- | --- | --- |
|  |  | Weight < 50 kg | Weight ≥ 50 kg |  |
| Modern chemotherapeutic drugs | | | |  |
| Amikacin (Am) | Injection | 0.4 g (once a day) | 0.6 g (once a day) | Yi Chang Humanwell Pharmaceutical Co.,Ltd. (Yichang, Hubei, China). |
| Moxifloxacin (Mfx) | Tablet | 0.4 g (once a day) | 0.4g (once a day) | Fuyuan Pharmaceutical Co., Ltd.(Beijing, China) |
| Cycloserine (Cs) | Capsule | 0.25 g (twice a day) | 0.25 g (twice a day) | Dong-A ST Co.,Ltd(Seoul, South Korea) |
| Promethionine (Pto) | Tablet | 0.3g (twice a day) | 0.4 g (twice a day) | Dr. Kang Pharmaceutical Co., Ltd.(Shenyang, Liaoning, China) |
| Pyrazinamide (Z) | Tablet | 1.5 g (once a day) | 1.5 g (once a day) | Hoingqi Pharmaceutical Co., Ltd.(Shenyang, Liaoning, China) |
| Ethambutol (E) | Tablet | 0.25g (twice a day) | 0.25 g (three times a day) | Hoingqi Pharmaceutical Co., Ltd.(Shenyang, Liaoning, China) |
| TCM compound |  |  |  |  |
| No.1 compound | granules | 8.2 g (twice a day) | 8.2 g (twice a day) | Neo-Green Pharmaceutical Technology Development Co., Ltd. (Chengdu, Sichuan, China) |
| No.2 compound | granules | 6.92 g (twice a day) | 6.92 g (twice a day) | Neo-Green Pharmaceutical Technology Development Co., Ltd. (Chengdu, Sichuan, China) |
| No.3 compound | granules | 6.6 g (twice a day) | 6.6 g (twice a day) | Neo-Green Pharmaceutical Technology Development Co., Ltd. (Chengdu, Sichuan, China) |
| No.4 compound | granules | 6.47 g (twice a day) | 6.47 g (twice a day) | Neo-Green Pharmaceutical Technology Development Co., Ltd.(Chengdu, Sichuan, China) |
| No.1 placebo | granules | 8.2 g (twice a day) | 8.2 g (twice a day) | Neo-Green Pharmaceutical Technology Development Co., (Chengdu, Sichuan, China) |
| No.2 placebo | granules | 6.92 g (twice a day) | 6.92 g (twice a day) | Neo-Green Pharmaceutical Technology Development Co., Ltd. (Chengdu, Sichuan, China) |
| No.3 placebo | granules | 6.6 g (twice a day) | 6.6 g (twice a day) | Neo-Green Pharmaceutical Technology Development Co., Ltd. (Chengdu, Sichuan, China) |
| No.4 placebo | granules | 6.47g (twice a day) | 6.47 g (twice a day) | Neo-Green Pharmaceutical Technology Development Co., Ltd. (Chengdu, Sichuan, China) |

Table 4 Evaluation criteria of traditional Chinese medicine syndrome score in MDR-PTB

| Symptoms | Normal (0) | Mild（1） | Moderate（2） | Severe（3） |
| --- | --- | --- | --- | --- |
| Cough | Not significant | during the day only | Occasionally at any time | Frequently |
| Phlegm | Not significant | 10-50ml in 24 hours | 50-100ml in 24 hours | ＞100ml in 24 hours |
| Chest pain | Not significant | dull pain | Occasional pain | Obvious and frequent pain |
| Afternoon fever | Not significant | The body is not too hot to touch, and the fever duration is short | Touching the body heats the hands, and the fever lasts long | Touching the body like burnt charcoal, and the fever lasts longer |
| Hemoptysis | Not significant | Bloodshot in sputum | ≤100ml within 24h or once | ＞100ml within 24h or once |
| Flushed cheeks | Not significant | Slightly reddish | Obviously but not brilliant red | Red as makeup |
| Night sweat | Not significant | Occasional, mainly head and face | Recurring, wet chest and back | Frequently, wet all over the body |
| Dry throat | Not significant | Occasionally | often | All the time |
| Spontaneous sweat | Not significant | Occasionally, not much sweat | Recurring, sweating with little activity | Frequently, sweating without activity and a lot, even wet through underwear |
| Fatigue | Not significant | Can't work long hours but fatigue can recover in a short time | Feeling tired after a little labor with slow recovery | Feeling tired without work |
| Shortness of breath | Not significant | Shortness of breath after general physical activity | Shortness of breath after a little activity | Shortness of breath without activity |

Table 5 The follow-up process and time point for each subject in the study

|  | Study period | | | | | | |
| --- | --- | --- | --- | --- | --- | --- | --- |
|  | Enrolment | Allocation | Intervention | | | | |
| Timepoint* | -2 month | month 0 | month 1 | month 2 | month 3 | month 6 | month 11 |
| Enrolment: |  |  |  |  |  |  |  |
| Inclusion and exclusion criteria | √ |  |  |  |  |  |  |
| Informed consent | √ |  |  |  |  |  |  |
| Basic information | √ |  |  |  |  |  |  |
| Allocation |  | √ |  |  |  |  |  |
| Intervention: |  |  |  |  |  |  |  |
| Shorter regimen+ No.1+ NO.3 compound |  |  |  |  |  |  |  |
| Shorter regimen+ No.1+ No. 3 placebo |  |  |  |  |  |  |  |
| Shorter regimen+ No.2+No.4 compound |  |  |  |  |  |  |  |
| Shorter regimen+ No.2+No.4 placebo |  |  |  |  |  |  |  |
| Outcome measure |  |  |  |  |  |  |  |
| Cure rate |  |  |  |  |  |  | √ |
| Sputum-culture conversion | √ |  | once a week | | | Once a month | |
| Lesion absorption change | √ |  |  | √ |  | √ | √ |
| Cavity closure change | √ |  |  | √ |  | √ | √ |
| TCM symptom score | √ |  | Once a month | | | | |
| Safety assessments: |  |  |  |  |  |  |  |
| Adverse events recorded | √ |  |  |  |  |  |  |
| Physical examination | √ |  |  |  |  |  |  |
| Blood and urine routine | √ |  | Once a month | | | | |
| Blood glucose | √ |  | Once a month | | | | |
| Liver and Kidney function | √ |  | Once a month | | | | |
| Electrocardiogram (ECG) | √ |  | Once a month | | | | |

**Part 2**

This appendix has been provided by the authors to give readers additional information about this work.

**ABBREVIATIONS**

ULN = Upper Limit of Normal

LLN = Lower Limit of Normal

Rx = Therapy

Req = Required

Mod = Moderate

IV = Intravenous

ADL = Activities of Daily Living

Dec = Decreased

**ESTIMATING SEVERITY GRADE**

For abnormalities NOT found elsewhere in the Toxicity Tables use the scale below to estimate grade of severity:

| GRADE 1 | Mild | Transient or mild discomfort (< 48 hours); no medical intervention/therapy required. |
| --- | --- | --- |
| GRADE 2 | Moderate | Mild to moderate limitation in activity; some assistance may be needed; no or minimal medical intervention/therapy required. |
| GRADE 3 | Severe | Marked limitation in activity; some assistance usually required; medicalintervention/therapy required, hospitalizations possible |
| GRADE 4 | Potentially  Life threatening | Extreme limitation in activity; significant assistance required; significant medical intervention/ therapy required, hospitalization or hospice care probable. |

**SERIOUS OR LIFE-THREATENING AEsU**

ANY clinical event deemed by the clinician to be serious or life-threatening should be considered a grade 4 event. Clinical events considered to be serious or life-threatening include, but are not limited to: seizures, coma, tetany, diabetic ketoacidosis, disseminated intravascular coagulation, diffuse petechiae, paralysis, acute psychosis, severe depression.

**COMMENTS REGARDING THE USE OF THESE TABLES**

1. Standardized and commonly used toxicity tables ( World Health Organization [WHO]) have been adapted for use by the Division of Microbiology and Infectious Diseases (DMID) and modified to better meet the needs of participants in DMID trials.
2. For parameters not included in the following Toxicity Tables, sites should refer to the “Guide

For Estimating Severity Grade”.

1. Criteria are generally grouped by body system.
2. Some protocols may have additional protocol specific grading criteria, which will supercede the use of these tables for specified criteria.

| **PARAMETER** | **GRADE 1** | **GRADE 2** | **GRADE3** | **GRADE4** |
| --- | --- | --- | --- | --- |
| **HEMATOLOGY** |  |  |  |  |
| Hemoglobin | 9.5-105 gm/dL | 8.0-9.4 gm/dL | 6.5-7.9 gm/dL | < 6.5 gm/dL |
| Absolute Neutrophil Count | 1000-1500mm^3^ | 750 - 999/mm^3^ | 500-749/mm^3^ | <500 mm^3^ |
| Platelets | 75000-99999/mm^3^ | 50000 - 74999/mm^3^ | 20000-49999/mm^3^ | < 20000/mm^3^ |
| White Blood Cells (WBCs)^1^ | 10000-12999/mm^3^  or 1000 – 3999/mm³ | 13000-15000/mm^3^ | 15000 - 30000/mm^3^ | >30000 mm3 or < 1000 mm3 |
| % Polymorphonuclear Leucocytes + Band Cells | >80-89% | 90-95% | >95% | — |
| Abnormal Fibnnogcn | Low: 100 - 200mg/dL  High: 400 - 600mg/dL | Low: < 100 mg/dL  High: >600 mg/dL | Low: < 50 mg/dL | fibrinogen associated with gross bleeding or with disseminated coagulation |
| Fibrin Split Product | 20- 40 mcg/mL | 41-50 mcg/mL | 51-60 mcg/mL | >60 mcg/mL |
| Prothrombin Time (PT) | > 1.00 to 1.25 x ULN | >1.25 to <1.30 x ULN | >1.30 to<3.00 x ULN | > 3.00 x ULN |
| Activated Partial Thromboplastin (APPT) | >1.00 to <1.66 x ULN | >1.66 to <2.33 x ULN | >2.33 to <3.00 x ULN | >3.00 x ULN |
| Methemoglobin | 5.0-9.9 % | 10.0-14.9% | 15.0-19.9% | >20.0% |

| **PARAMETER** | **GRADE 1** | **GRADE2** | **GRADE 3** | **GRADE 4** |
| --- | --- | --- | --- | --- |
| **CHEMISTRIES** | | | | |
| Hyponatremia | 130 - 135 mEq/L | 123 - 129 mEq/L | 116 - 122 mEq/L | < 116 mEq/L or abnormal sodium with mental status changes or seizures |
| Hypernatremia | 146 - 150 mEq/L | 151 - 157 mEq/L | 158 - 165 mEq/L | > 165 mEq/L or abnormal sodium with mental status changes or seizures |
| Hypokalemia | 3.0 - 3.4 mEq/L | 2.5 - 2.9 mEq/L | 2.0 -2.4 mEq/L or intensive replacement therapy or hospitalization require d | < 2.0 mEq/L or abnormal potassium with paresis, ileus or hfe-threalening arrhythmia |
| Hyperkalemia | 5.6 - 6.0 mEq/L | 6.1 - 6.5 mEq/L | 6.6 - 7.0 mEq/L | > 7.0 mEq/L or abnormal potassium with hfe-threatening arrhythmia |
| Hypoglycemia | 55 - 64 mg/dL | 40 - 54 mg/dL | 30 - 39 mg/dL | < 30 mg/dL or abnormal glucose with mental status changes or coma |
| Hyperglycemia (nonfasting and no prior diabetes) | 116 - 160 mg/dL | 161-250 mg/dL | 251-500 mg/dL | > 500 mg/dL or abnormal glucose with ketoacidosis or seizures |
| Hypocalcemia (corrected for albumin) | 8.4 - 7.8 mg/dL | 7.7-7.0 mg/dL | 6.9 - 6.1 mg/dL | <6.1 mg/dL or abnormal calcium with hfe threatening arrhythmia or tetany |
| Hypomagnesemia | 1.4 - 1.2 mEq/L | 1.1 -0.9 mEq/L | 0.8 - 0.6 mEq/L | < 0.6 mEq/L or abnormal magnesium with hfe-threatening arrhythmia |
| Hypophosphatemia | 2.0-2.4 mg/dL | 1.5- 1.9 mg/dL or re pl ace meni Rx required | 1.0 - 1.4 mg/dL intensive Rx or hospitalization required | < 1.0 mg/dL or abnormal phosphate with hfe-threatening arrhythmia |
| Ilyperbihrubi nemia | > 1.00 to ≤ 1.50 x ULN | > 1.50 to ≤2.50 x ULN | >2.50 to ≤5.00 x ULN | > 5.00 x ULN |
| BUN | > 1.24 to ≤ 2.50 x ULN | >2.50 to ≤5.00 x ULN | >5.00 to ≤ 10.00 x ULN | > 10.00 x ULN |
| Hyperuricemia (uric acid) | 7.5 - 10.0 mg/dL | 10.1 - 12.0 mg/dL | 12.1 - 15.0 mg/dL | > 15.0 mg/dL |
| Creatinine | >1.00to ≤1.50 x ULN | > 1.50 to ≤ 3.00 xULN | >3.00 to ≤ 6.00 x ULN | > 6.00 x ULN or dialysis required |

| **PARAMETER** | **GRADE 1** | **GRADE2** | **GRADE 3** | **GRADE 4** |
| --- | --- | --- | --- | --- |
| **RESPIRATORY** | | | | |
| Cough | transient;  no treatment required | persistent cough;  treatment responsive | Paroxysmal cough;  uncontrolled with  treatment | - |
| Bronchospasm,  Acute | transient;  no treatment required;  FEV1 70% – 80% of  peak flow | requires treatment;  normalizes with  bronchodilator; FEV1 50% – 70% of  peak flow | no normalization with  bronchodilator;  FEV1 25% – 50% of  peak flow or retractions present | cyanosis: FEV1 < 25% of peak  flow or intubation necessary |
| Dyspnea | dyspnea on exertion | dyspnea with normal  activity | dyspnea at rest | dyspnea requiring oxygen  Rx |
| **GASTROINTESTINAL** | | | | |
| Nausea | mild or transient; maintains reasonable intake | moderate discomfort; intake decreased significantly; some activity limited | no signifkant intake; requires IV fluids | liospitalization required |
| Vomiting | 1 episode in 24 hours | 2-5 episodes in 24 hours | > 6 episodes in 24 hours or needing IV fluids | physiologic consequences requiring hospitalizalion or requiring parenteral nutrition |
| Constipation | requiring stool softener or dietary modification | requiring laxatives | obstipation requiring manual evacuation or enema | obstruction or toxic megacolon |
| Diarrhea | mild or transient; 3-4 loose stools/day or mild diarrheal last < 1 week | moderate or persistent; 5-7 loose stools/day or diarrheal lasting > 1 week | > 7 loose stool s/day; or bloody diarrhea; or orthostatic hypotension; or electrolyte imbalance; or>2L IVfluids required | hypotensive shock or physiologic consequences requiring hospitalisation |
| Oral Discomfort /Dysphagia | mild discomfort; no difficulty swallowing | some limits on eating/drinking | eating/talking very limited; unable to swallow solid foods | unable to drink fluids; requires IV fluids |

| **PARAMETER** | **GRADE 1** | **GRADE 2** | **GRADE3** | **GRADE 4** |
| --- | --- | --- | --- | --- |
| **NEUROLOGICAL** | | | | |
| Neuro-Cerebellar | shght incoordination dysdiadochokinesis | intention tremor, dysmetria, slurred speech; nystagmus | locomotor ataxia | incapacitated |
| Psjchiatric | mild anxiety or depression | moderate anxiety or depression; Rx required; change in normal routine | severe mood changes requiring Rx; or suicidal ideation; or aggressive ideation | acute psychosis requiring hospitalization; or suicidal geslure/attempt or lulluci nations |
| Muscle Strength | subjective weakness no objective symptoms/ signs | mild objective signs/symptoms no decrease in function | objective weakness function limited | paralysis |
| Paresthesia (burning, tingl ing_5_ etc.) | mild discomfort; no treatment required | moderate discomfort; non-narcotic analgesia required | severe discomfort; or narcotic analgesia required with symptomatic improvement | incapacitating; or nol responsixe to narcotic analgesia |
| Neuro-sensor | mild impairment in sensation (decreased sensation, e.g., vibrator), pinprick, hot/cold in great toes) in fbcal area or symmetrical distribution; or change in taste, smell, vision and/or hearing | moderate impairment (mod decreased sensation, e.g., vibrator)/pinprick, hot/cold to ankles) and/or joint position or mild impairment tliat ts nol symmetrical | severe impairment (decreased or loss of sensation to knees or wrists) or loss of sensation of at least mod degree in multiple diftereni body areas (ie, upper and lower extremities) | sensory loss involves hmbs and trunk; or paralysis; or seizures |
| **MUSCULOSKELETAL** | | | | |
| Arthralgia (joint pain) | mild pain not interfering  with function | moderate pain, analgesics and /or pain interfering with function but not with activities of daily living | severe pain; pain and/or  analgesics interfering with activities of daily living | disabling pain |
| Arthritis | mild pain with  inflammation, erythema or joint swelling – but not interfering with function | moderate pain with inflammation,  erythema or joint swelling-interfering with function, but not with activities of daily living | severe pain with inflammation,  erythema or joint swelling – and interfering with activities of daily living | permanent and/or  disabling joint distruction |
| Myalgia | myalgia with no  limitation of activity | muscle tenderness (at other than injection site) or with moderate impairment of activity | severe muscle tenderness  with marked impairment of activity | frank myonecrosis |

| **PARAMETER** | **GRADE 1** | **GRADE 2** | **GRADE3** | **GRADE 4** |
| --- | --- | --- | --- | --- |
| **SKIN** | | | | |
| Mucocutaneous | erythema; pruritus | diftuse, maculo papular rash, dry desquamation | vesiculation or moist desquamation or ulceration | exfoliative dermalitis, mucous membrane involvement or erythema, multifbrme or suspected Steve ns-Johnson or necrosis requiring surgery |
| Induration | < 15mm | 15 一 30 mm | > 30mm |  |
| Erjlhema | < 15mm | 15 一 30 mm | > 30mm |  |
| Edema | < 15mm | 15 一 30 mm | > 30mm |  |
| Rash at Ityection Sue | < 15mm | 15 一 30 mm | > 30mm |  |
| Pruritus | slight itching at injection site | moderate itching at injection extremity | itching over entire body |  |
| **SYSTEMIC** | | | | |
| Allergic Reaction | pruritus without rash | localized urticaria | generalized urticaria;  angioedema | anaphylaxis |
| Headache | mild, no treatment required | transient, moderate; treatment required | severe; responds lo initial narcotic tlierapy | intractable; requires repealed narcotic therapy |
| Fever(oral) | 37.7-38.5 ℃ | 38.6-39.5 ℃ | 39.6-40.5 ℃ | > 40.0 ℃ |
| Fatigue | normal activity reduced <48 hours | normal activity decreased 25- 50% >48 hours | normal activity decreased > 50% can't work | unable to care for self |

**Addendum 4: Cardiovascular Safety - Abnormalities**

**ECG***

All important abnormalities from the ECG readings will be reported. The following abnormalities will be defined for ECG parameters:

QTc (ms):

Absolute QTc interval prolongation: > 450; > 480; > 500.

Change from baseline in QTc interval:

QTc interval increase from baseline >30;

QTc interval increase from baseline >60;

QRS (ms): normal < 120 ≤ abnormal;

QTc change (ms): normal < 30 < borderline < 60 < abnormal high.

**Vital Signs**

The following abnormalities will be defined for vital signs:

Pulse (beats per minute):

abnormally high: ≥120 bpm;

abnormally low: ≤ 50 bpm.

DBP (mmHg):

abnormally high: Grade 1 or mild: > 90 to ≤ 100 mmHg; Grade 2 or moderate: > 100 to ≤ 110 mmHg; Grade 3 or severe: > 110 mmHg;

abnormally low: ≤ 50 mmHg

SBP (mmHg):

abnormally high: Grade 1 or mild: > 140 to ≤ 160 mmHg; Grade 2 or moderate: > 160 to ≤ 180 mmHg; Grade 3 or severe: > 180 mmHg.

abnormally low: ≤ 50 mmHg.

Systemic reaction: Allergic Reaction, Headache, Fever, Fatigue.

Laboratory tests: Elevated erythrocyte, Lower hemoglobin, Elevated hemoglobin, Lower leukocyte, Elevated leukocyte, Elevated platelet, Lower platelet, Elevated uric acid, Elevated urinary red blood cells, Urine leukocytosis, Elevated urinary protein, Elevated urine, Elevated alanine aminotransferase, Elevated aspartate aminotransferase, Lower total bilirubin, Elevated total bilirubin, Lower blood urea nitrogen, Elevated blood urea nitrogen, Lower creatinine, Elevated creatinine.

Respiratory system: Upper respiratory tract infection, Spontaneous, Lung infection, Lung inflammation

Digestive system: Vomit, Impaired liver function, Nausea, Bloating, Gastrointestinal discomfort, Constipation, Diarrhea, Oral Discomfort, Dysphagia

Skin System: Mucocutaneous, Induration, Erjlhema, Edema, Rash at Ityection, Pruritus

Neurological system: Neuro-cerebellar, Psjchiatric, Muscle Strength, Paresthesia (burning, tingling), Neuro-sensor.

Musculoskeletal: Arthralgia (joint pain), Arthritis, Myalgia

**References**

Diacon AH, Pym A, Grobusch MP, de los Rios JM, Gotuzzo E, Vasilyeva I, Leimane V, Andries K, Bakare N, De Marez T, Haxaire-Theeuwes M, Lounis N, Meyvisch P, De Paepe E, van Heeswijk RP, Dannemann B; TMC207-C208 Study Group. Multidrug-resistant tuberculosis and culture conversion with bedaquiline. N Engl J Med. 2014 Aug 21;371(8):723-32. doi: 10.1056/NEJMoa1313865.
